# Editorial: Nutrient use efficiency of plants under abiotic stress

**DOI:** 10.3389/fpls.2023.1179842

**Published:** 2023-05-26

**Authors:** Bo-Wen Liang, Chao Li, Tuan-Hui Bai, Ping Wang

**Affiliations:** ^1^ College of Horticulture, Hebei Agricultural University, Baoding, Hebei, China; ^2^ State Key Laboratory of Crop Stress Biology for Arid Areas/Shaanxi Key Laboratory of Apple, College of Horticulture, Northwest A & F University, Yangling, China; ^3^ College of Horticulture, Henan Agricultural University, Zhengzhou, Henan, China; ^4^ Department of Genetics, Development and Cell Biology, Iowa State University, Ames, IA, United States

**Keywords:** plants, abiotic stress, nutrient use efficiency (NUE), melatonin, plant resistance

Abiotic plant stresses such as drought, flooding, and ultraviolet (UV) radiation have intensified in recent decades due to global climate change. Abiotic stress can result in fundamental changes to cellular processes and whole-plant physiology that allow the plant to adapt to the environment (Wang et al., 2021). Mineral nutrients play electrochemical, structural, and catalytic roles in all biological organisms, and are essential for the completion of plant life cycle (Lopez et al., 2023). Abiotic stresses and nutrient deficiency severely impact the growth, development, and productivity of plants (Shikha et al., 2023). Environmental changes cause abiotic stress in plants primarily by alterations in the uptake and utilization of the nutrients. Maintaining nutrient use efficiency under abiotic stress is an effective means of increasing plant stress resistance. Thus, the intensification of abiotic stresses will require the development of plants with high nutrient use efficiency. There have been effects to increase plant abiotic stress tolerance or growth with application small molecules, melatonin is such a molecule. Exogenous melatonin application has been shown to effectively increase stress tolerance and nutrient uptake in plants, and other compounds also play key roles in nutrient uptake under abiotic stress conditions (Zhang et al., 2015; Liu et al., 2020a; Sun et al., 2021; Gao et al., 2022; Ahammed and Li, 2023)

In this Research Topic, we present 11 articles related to abiotic stress responses and nutrient use efficiency in plants, with a focus on the relevant factors that influence these processes. Although abiotic stresses and nutrient deficiency can limit plant growth and survival, plants have evolved a unique set of complex mechanisms to cope with environments under high climate variation. (Liu et al., 2022; Wang et al., 2022; Abiala et al., 2023). Therefore, research related to physiological, biochemical, and molecular responses, as well as nutrient uptake and utilization in plants, is of paramount importance to improve plant stress responses and nutrient use efficiency. Yue et al. integrated envirotyping techniques and multi-trait selection to enhance the mean performance and stability of maize genotypes, opening the door to more systematic and dynamic characterization of the environment to better understand genotype-by-environment interactions in multi-environment trials. Sun et al. provided new insights into the molecular mechanism of the iron deficiency response in *Malus baccata*. This study revealed that *MbHY5-MbYSL7* mediates chlorophyll synthesis and iron transport under iron-deficient conditions. Δ1-Pyrroline-5-carboxylate synthetase (P5CS) is the rate-limiting enzyme in proline biosynthesis, and plays an essential role in plant responses to environmental stresses. Ma et al. identified 11 *PbP5CS* genes in pear trees, most of which exhibited distinct expression patterns in response to drought, waterlogging, salinity/alkalinity, and other abiotic stresses. These findings represent an advance in the understanding of the physiological functions of *PbP5CS* genes in the enhancement of stress tolerance in pear and other fruit trees. Song et al. performed transcriptome deep sequencing and weighted gene co-expression network analyses to explore the molecular mechanism of blueberry calli in response to UV-B radiation. They found that UV-B induced the expression of flavonoid biosynthetic pathways, and suggested that direct or indirect regulation of MYB inhibitors or activators promotes flavonoid biosynthesis under UV-B radiation. In a review article, Du et al. highlighted the structure and function of TIR1/AFB family members, with an emphasis on the potential mechanisms by which these proteins regulate abiotic stress responses at the transcriptional and post-transcriptional levels, including downstream regulation. For example, they may function in the drought tolerance, salt stress, and nitrate stress pathways. Chalcone synthase (CHS) is a key enzyme required in flavonoid synthesis. Liu et al. isolated a CHS gene from *Poncirus trifoliata* and found that relative expression of the *PtCHS* gene was regulated by soil water deficit and arbuscular mycorrhizal fungi (AMF) inoculation. Xu et al. explored the physiological roles of *CgSTPs* in pummelo, and found that *CgSTP4* plays important roles in sugar accumulation and pollen tube growth. Sun et al. compared the results of physiological, transcriptome, and metabolite analyses under different potassium conditions in apple seedlings. They found that apple seedlings regulate the carbon metabolism and flavonoid pathways in response to low and high potassium stress. This study provided new insights that may be used to improve potassium utilization efficiency in apple trees.

Melatonin is found in almost all plant tissues, and is powerful natural antioxidants that play a significant role in enhancing plant tolerance to various abiotic stressors such as drought (Muhammad et al., 2023), flooding (Moustafa-Farag et al., 2020), salt (Michard and Simon, 2020), and nutrient deficiency (Cao et al., 2022a). Exogenous application of other compounds also enhances plant abiotic stress resistance and nutrient utilization efficiency. The benefits of dopamine have been reported in previous studies on water-induced stress, which showed that exogenous dopamine enhances the tolerance of drought (Du et al., 2022a) and waterlogging (Cao et al., 2022b) by apple trees by regulating the rhizosphere microbiome. Previous studies have also reported that melatonin and dopamine significantly improve plant nutrient use efficiency (Liu et al., 2020b; Du et al., 2022b). Ionome nutrient uptake was decreased in drought-stressed plants, whereas exogenous melatonin and dopamine significantly increased the uptake of mineral elements, particularly under drought stress conditions (Liang et al., 2018a; Liang et al., 2018b). In this Research Topic, Huo et al. reported that exogenous melatonin effectively alleviated damage to kiwifruit plants in response to waterlogging stress. This study provides new insights into the links between melatonin and amino acid metabolic systems in plant stress tolerance. Xia et al. evaluated the effects of melatonin and AMF on kiwifruit seedling drought tolerance. They found that melatonin and AMF have a synergistic effect on improving drought tolerance by increasing mycorrhizal colonization and nutrient uptake. Ma et al. found that AMF (*Diversispora spurca*) promoted growth in walnut plants exposed to drought stress. Similarly, AMF increased apple tree drought resistance by regulating MAPK pathway genes (Huang et al., 2020). AMF is a useful tool for increasing plant nutrient uptake under drought stress conditions (Lotfabadi et al., 2022). Gao et al. (2020) reported that dopamine promoted AMF symbiosis by increasing carbohydrate content, and the synergistic effect of dopamine and AMF enhanced apple tree salt resistance.

Abiotic stresses are anticipated to be among the greatest challenges to future agriculture. It can diminish the uptake and utilization of elements, then influence plant nutrient status. Nutrient deficiency will continue to limit plant growth and yield. Most of the articles associated with this Research Topic increase our understanding of plant adaptive responses to abiotic stresses and nutrient use efficiency, and enrich current knowledge of the mechanisms through which melatonin and other compounds facilitate abiotic stress responses and nutrient utilization efficiency in plants, allowing them to adapt to unfavorable environmental conditions. These findings will offer new opportunities for its use in agriculture, especially in regions that are challenged by abiotic stress or nutrient deficiency condition. We hope that this Research Topic will inspire new ideas and stimulate further research in these fields.

## Author contributions

All authors listed have made a substantial, direct, and intellectual contribution to the work, and approved it for publication.
